# Tailoring of Hierarchical Porous Freeze Foam Structures

**DOI:** 10.3390/ma15030836

**Published:** 2022-01-22

**Authors:** David Werner, Johanna Maier, Nils Kaube, Vinzenz Geske, Thomas Behnisch, Matthias Ahlhelm, Tassilo Moritz, Alexander Michaelis, Maik Gude

**Affiliations:** 1 Fraunhofer Institute for Ceramic Technologies and Systems, IKTS, Winterbergstraße 28, 01277 Dresden, Germany; tassilo.moritz@ikts.fraunhofer.de (T.M.); alexander.michaelis@ikts.fraunhofer.de (A.M.); 2 Institute of Lightweight Engineering and Polymer Technology, University of Dresden, Holbeinstraße 3, 01307 Dresden, Germany; Vinzenz.Geske@tu-dresden.de (V.G.); Thomas.Behnisch@tu-dresden.de (T.B.); maik.gude@tu-dresden.de (M.G.); 3 Fraunhofer Institute for Ceramic Technologies and Systems, IKTS, Maria-Reiche-Str. 2, 01109 Dresden, Germany; nils.kaube@ikts.fraunhofer.de (N.K.); matthias.ahlhelm@ikts.fraunhofer.de (M.A.)

**Keywords:** freeze foaming, bioceramics, porous ceramics, ceramic foams, in-situ computed tomography, none destructive testing

## Abstract

Freeze foaming is a method to manufacture cellular ceramic scaffolds with a hierarchical porous structure. These so-called freeze foams are predestined for the use as bone replacement material because of their internal bone-like structure and biocompatibility. On the one hand, they consist of macrostructural foam cells which are formed by the expansion of gas inside the starting suspension. On the other hand, a porous microstructure inside the foam struts is formed during freezing and subsequent freeze drying of the foamed suspension. The aim of this work is to investigate for the first time the formation of macrostructure and microstructure separately depending on the composition of the suspension and the pressure reduction rate, by means of appropriate characterization methods for the different pore size ranges. Moreover, the foaming behavior itself was characterized by in-situ radiographical and computed tomography (CT) evaluation. As a result, it could be shown that it is possible to tune the macro- and microstructure separately with porosities of 49–74% related to the foam cells and 10–37% inside the struts.

## 1. Introduction

Ceramic foams cover a wide range of applications, including as a support material for catalysts [1,2,3], pore burners [4], and thermal insulators [5], as well for waste water treatment [6], metal filtration [7], and in scaffolds for bone substitute [8,9,10]. They can be manufactured, e.g., by the polymeric sponge method [11], direct foaming methods [12,13], or freeze casting [14,15,16]. Freeze foams are innovative cellular structures based on a direct foaming process manufactured with any material processable by powder technology. This freeze foaming process does not require the use of organic templates and pore formers and is particularly important for the production of ceramic foam structures. Possible applications cover a wide range from biomedical uses, e.g., artificial bones, support material for catalysts, pharmaceutical products, as well as thermal insulators [17,18,19]. The diversity of these applications results from the range of initial materials (ceramics, metals, metal-organic frameworks), variable starting suspensions, and the resulting foam structure properties. The latter is characterized, e.g., by the cell geometry, the cell size distribution, the proportions of open and closed cells, and the type of cell struts, which are formed either by pore-forming gases (air or steam) or by ice crystals.

The foam cell structure in freeze foaming is created by pressure-induced inflation of an aqueous, e.g., ceramic, suspension inside the vacuum chamber of a freeze dryer. This allows a subsequent pressure-controllable freezing upon crossing the triple point (referring to p,T-phase diagram), followed by freeze-drying of the resulting foam [20,21]. With decreasing ambient pressure, both the air entrapped during suspension production and the vapor produced by lowering the boiling point act as blowing agents for the occurring foam formation. During the further ambient pressure reduction, the temperature of the ceramic suspension follows the equilibrium line in the phase diagram of water in the direction of the triple point and the foaming increases. A further drop in vacuum pressure causes the equilibrium temperature of the suspension to fall below its freezing point at the triple point. This leads to immediate freezing and a resulting stabilization of the foam. The formed cellular structure subsequently freeze-dries. The dried foam is solidified by a heat treatment (sintering), forming its final ceramic properties. The ceramic foam structure has a comparatively high mechanical strength due to filled struts and shows a bimodal pore size distribution. The structure of a sintered freeze foam consists of two hierarchical levels. Pore formation by the expansion of entrapped air and released vapor leads to macro-structural foam cells while pore formation by the sublimation of ice crystals (replicas of the ice crystals) leads to micro-structural strut pores as well as freezing structures [22,23].

The foam production process is influenced by a complex interaction of various process and material parameters, which are selected empirically and have been difficult to reproduce so far. To be able to design the foam properties in a targeted and application-related manner, it was necessary to analyze the foam formation process and the effect of the different components of the suspension, as well as the process parameters like reduction rate on structural properties.

In the initial investigation, it was possible to gain a deeper understanding of the principal mechanisms relating to the formation of freeze structures by carrying out analyses of the influence of selected suspension and process parameters on the resulting structural properties of biocompatible ceramic foams [22,23,24,25]. With the help of modern methods of non-destructive material testing, a first-time phenomenological investigation and foam structure analysis during the foaming process could be carried out by using in-situ X-ray radiography. As a result, three essential pore formation factors were identified: air and water content/vapor, suspension temperature, and pressure reduction rate. The temperature-dependent foaming behavior is confirmed on the basis of X-ray radiographic and computer tomographic investigation. In particular, the pressure reduction rate has a demonstrable influence on the shape of the formed pores. 

Based on the knowledge gained, the focus of this study lies on the targeted adjustment and tailoring of the macrostructure (homogenization of the pore size/distribution in the foam cells) and the microstructure (influencing and controlling the freezing structures in the strut pores) to create stress- and application-adapted ceramic foams. A stable and shrinkage-adaptable freeze foaming process is developed by investigating the influence of composition of the suspension (water, binder, and thickener contents) on the resulting foam structure and additionally by a controlled adjustment of pressure reduction.

## 2. Materials and Methods

### 2.1. Design of Experiments (DoE)

**Figure 1:**
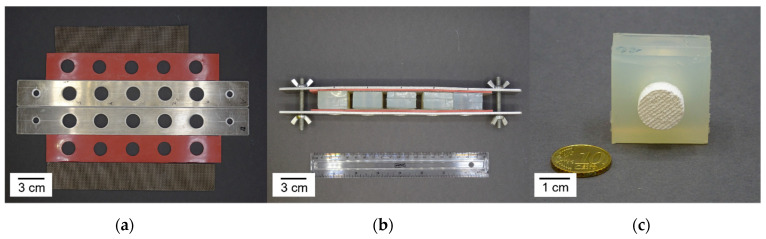
(**a**) Frame for holding rubber molds and sieves closing on both sides. (**b**) rubber molds fixed inside the frame. (**c**) dried freeze foam inside rubber mold.

The aim of this study was to determine the influence of the suspension composition and its resulting rheological behavior on the hierarchical porous foam structure consisting of foam cells and strut pores. As ceramic raw material, hydroxyapatite (HAp) (Merck KGaA, Darmstadt, Germany; BET = 70 m^2^/g, d50 = 2.64 µm) was chosen. Prior to the preparation of suspension, the HAp powder was calcined at 900 °C for 2 h to reduce BET-surface to 6.7 m^2^/g. The calcined HAp was dispersed in water with 4.6 wt.% DOLAPIX CE64 (Co. Zschimmer & Schwarz Mohsdorf GmbH & Co. KG, Burgstädt, Germany) in relation to HAp and polyvinyl alcohol as a binder in a centrifugal vacuum mixer. In a second mixing step, a thickener (TAFIGEL AP15, Co. Münzing Chemie GmbH, Heilbronn, Germany) combined with 2-Amino-2-methylpropanol (AMP) was added. AMP shifts the pH-value to 8–10 so that the rheological modifier can display its full thickening capacity. The thickener TAFIGEL AP15 is a hydrophobically modified alkali swellable emulsion (HASE) on polyacrylate basis. The prepared suspension was filled into cylindrical rubber molds (Ø = 14 mm, h = 20 mm). After filling, the molds were closed with a sieve from both sides to ensure a defined outer foam geometry by allowing the pore forming gases, air and water vapor to escape (Figure 1). This is necessary to stop foam cell growth when cell walls tear apart.

**Figure 2:**
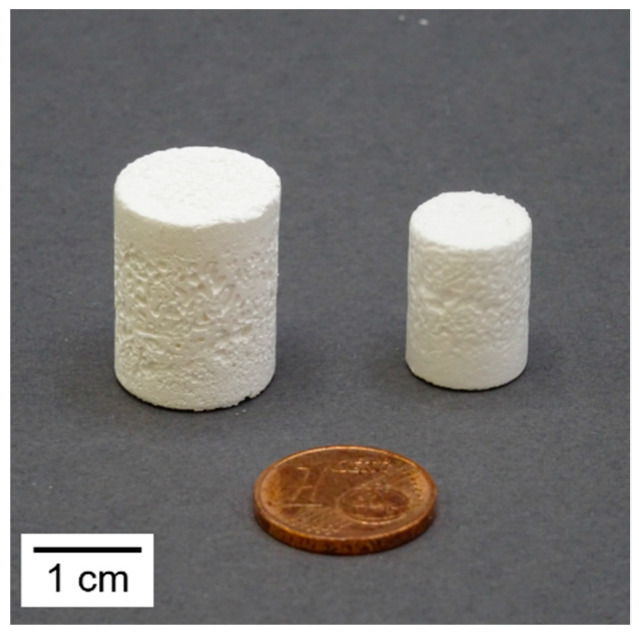
Left: green Freeze Foam, right: sintered Freeze Foam.

The filled molds were put in a freeze dryer (ALPHA 2-4 LSCPLUS, Co. Martin Christ Gefriertrocknungsanlagen GmbH, Osterode, Germany) at room temperature followed by pressure reduction from ambient pressure to 0.1 mbar in 2 min. After 6 h main drying, the pressure was further reduced to 0.001 mbar for final drying. The obtained green foams were debindered and sintered at 1280 °C for 1 h. Figure 2 shows a freeze foam in green state and after sintering for comparison.

A randomized two-stage full factorial test plan consisting of three factors and a three-time repetition of the central point was established (Table 1). Suspensions were prepared with 32 wt.% and 48 wt.% water contents. Moreover, PVA-binder and thickener content were varied according to the following equations:

*ω*_binder_ = *m*_binder_/(*m*_binder_ + *m*_HAp_) (1)

*ω*_thickener_ = *m*_thickener_/(*m*_thickener_ + *m*_water_) (2)

The DoE was evaluated regarding viscosity, foam cell porosity (*FCP*), and strut porosity (*StP*). To establish statistical models for these target values, the software Minitab v. 19.1 (Minitab Inc., München, Germany) was used. Variance analysis was carried out with a level of significance of α = 0.1. To simplify the models, backwards elimination was used to exclude insignificant terms.

### 2.2. Characterization Methods for DoE Evaluation

Rheological characterization of the suspension was conducted with a MODULAR COMPACT RHEOMETER MCR 302 from Anton Paar (Anton Paar Germany GmbH, Graz, Austria) equipped with a plate-plate system. Suspensions were measured with a regular shear test and shear rates ranging from 0.01 to 100 s^−1^. For evaluation, the viscosity at 10 s^−1^ was used. 

CT-analysis was conducted with a CT COMPACT (Co. Procon-X-Ray, Sarstedt, Germany) equipped with a flat panel detector. Scanning parameters were set to an acceleration voltage of 110 kV and a beam current of 100 µA. The resulting voxel size was 28.3 µm. Hence, 3D-analysis was performed with VGSTUDIO MAX V. 3.0 (Volume Graphics GmbH, Heidelberg, Germany). A detailed description of the porosity analysis can be found in a previous publication of the authors [23]. 

Strut porosity was determined by using an approach of Dammler et al. [26]:

*StP* = 1 − (*m*_foam_/*V*_struts_)/*ρ*_bulk_ (3)

Sintered foams were weighed and subsequently analyzed in the CT. Strut pores are smaller than the voxel size and therefore not detected. Consequently, the material volume of the scanned foams represents the strut volume *V*_strut_ including strut pores. The quotient of foam mass *m*_foam_ and strut volume is therefore equal to the geometrical density of the foam struts. Bulk density was measured with helium pycnometry on a crushed foam after sintering. Mercury porosimetry (AUTOPORE V, Co. Micromeritics Instrument Corporation, Norcross, GA, USA) and FESEM (ULTRA 55, Co. Carl Zeiss, Oberkochen, Germany) were chosen for analyzing the microstructure. The detectable pore size reaches from 4 nm to 400 µm, making this approach suitable for the identification of strut pore sizes. 

To determine the morphology of strut pores, FESEM was used. Sintered foams were embedded in epoxy resin, ceramographically prepared, and investigated with backscattered electrons to create a material contrast between ceramic and pores filled with epoxy resin.

### 2.3. Radiographical and CT Evaluation of a Model Suspension

**Figure 3:**
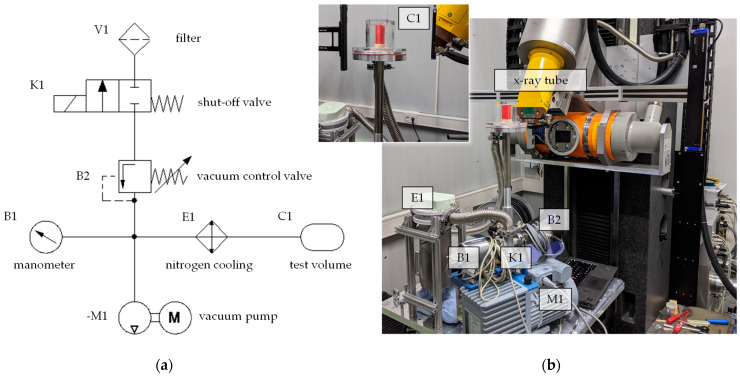
Test setup: (**a**) schematic diagram (**b**) installation in the in-situ radiographical and computed tomography.

Non-destructive X-ray inspection methods such as computed tomography (CT) offer a unique combination of advantages providing an insight into sections and samples of different materials and components. With current methods, material states can only be analyzed either before or after a change in the material [27,28]. An in-situ freeze foaming examination device, to be used inside a V|TOME|X L450 (General Electric, Cincinnati, OH, USA), was developed during former research activities, to investigate the freeze foaming during the process [25]. For the present experimental study, a modification of the developed in-situ CT device was required to ensure a quicker response of the vacuum system as well as an increase in either contrast or resolution [25]. The volume of the recipient being evacuated was decreased and an additional cold trap was installed. Combining those measures with an overall shortened vacuum tubing allowed higher pressure-reduction rates as well as improved responsiveness and repeatability. Furthermore, new components were added to the vacuum system’s feedback control (B1; B2) and an optimization of the software controller was implemented to monitor the pressure during the entire process (Figure 3a).

**Figure 4:**
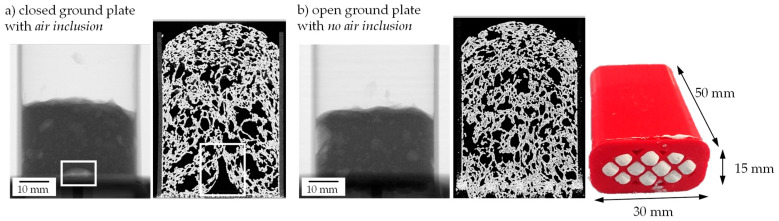
Comparison of foam structure after Freeze Foaming with a closed and open ground plate.

By increasing the height of the specimen chamber above the baseplate which holds the remaining device components, the distance between the tube and the center of the sample (FOD—focus/object distance) could be further decreased for a better resolution (Figure 3b). Since the focus of the examination lies in the quantification and investigation of the size distribution of pores, a decision was made to opt for better contrast rather than increased resolution. To this end, the reduced FOD was combined with a shorter distance between tube and detector, leading to higher intensity and enhanced contrast. As a result, smoother and more pronounced peaks in the histogram of volumes’ grey values allow for improved quality and reliability of the automatic material separation and defect analysis. Lastly, the design of 3D-printed specimen molds made from thermoplastic polyurethane (TPU) by a commercial FDM printer (Fused Deposition Modelling) was changed, now including an open ground plate to allow excess material to escape downwards without affecting the upper region of the foam too much (e.g., densification effects) (Figure 4).

X ray parameters were set to an acceleration voltage of 100 kV and a beam current of 300 µA on the 300 kV micro-focus X-ray tube, while images from the GE DXR-300 FLAT-BED DETECTOR were recorded using the highest sensitivity setting and an exposure time of 333 ms.

Two types of scans were performed: After filling molds with the model suspension (11.00 g; filling level 1/3), the freeze foaming process was started and recorded without rotation as a sequence of 2D radiographic images to observe the formation and coalescence of pores and measure the foaming speed as well as the final height. This was achieved by pixel tracking of the single images with the software IMAGEJ, as further described by Maier et al. [25]. 

Once the foaming stopped, the shut-off valve K1 was closed (Figure 3) and the structure was stabilized by decreasing the pressure below 6 mbar, the equilibrium pressure of the triple point of water. Subsequently, the final state was examined by conducting a CT scan with 720 projections and continuous rotation. Not only did the so-called fast-CT bring the scan duration down to 180 s, but the lack of accelerating and stopping reduced the risk of foam collapsing during the scan.

As mentioned before, the distance between tube and detector was lowered from 220 to 155 mm and the FOD was decreased from 2000 to 1100 mm. As a result, the resolution changed slightly from 22 to about 28 µm/vx, while the X-ray intensity on the detector increased by a factor of 2.4, greatly improving the contrast.

## 3. Results and Discussion

### 3.1. Viscosity

**Figure 5:**
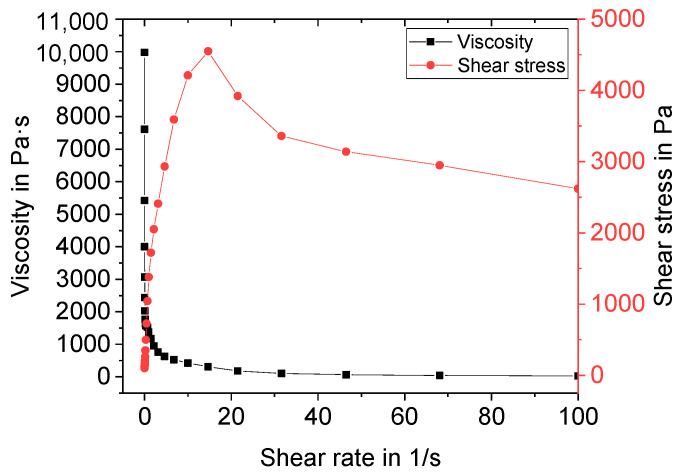
Exemplary flow curve and viscosity of suspension Z5.

Manufactured suspensions were characterized regarding their rheological behavior before freeze foaming. A standard shear test with shear rates ranging from 0.01 to 100 s^−1^ was performed. An exemplary flow curve and the viscosity of Z5 (central point) are depicted in Figure 5.

**Figure 6:**
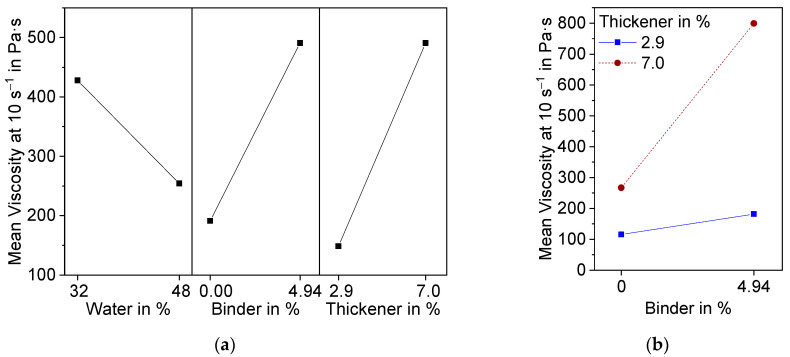
Effect plots of suspension viscosity at a shear rate of 10 s^−1^ resulting from DoE; (**a**) Main effects plot; (**b**) Interaction effect plot.

The suspension shows shear thinning behavior. Above a shear rate of 10 s^−1^, the shear stress suddenly decreases. This can be attributed to a lack of adhesion of the suspension to the plate. Therefore, slippage occurred at higher shear rates. For evaluation of the DoE, the viscosity at 10 s^−1^ was used. The results are presented in Figure 6 as a main effect plot (a) and interaction plot (b). The main effects plot shows the mean values of two different levels of the factors water, binder, and thickener contents. All three factors are, according to variance analysis in combination with backwards elimination, statistically significant (α < 0.1).

As anticipated, an increase in water content caused the viscosity of the suspension to decrease, whereas binder and thickener raised the viscosity. Additionally, binder and thickener showed significant interaction effects (Figure 6b). At 2.9 wt.% thickener, there was only a small increase in viscosity due to the addition of binder, while the increase was significantly higher at 7.0 wt.% thickener. This can be attributed to the associative thickening effect between TAFIGEL AP15 and the PVA used as binder [29].

**Figure 7:**
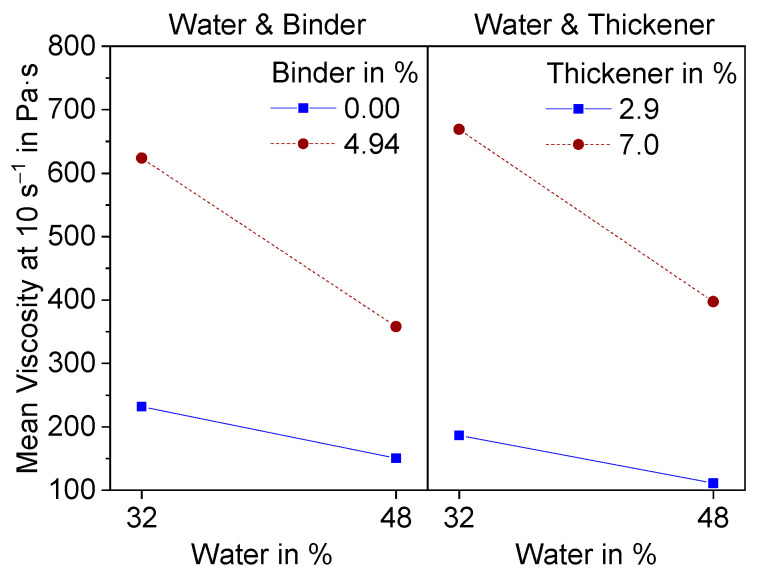
Interaction effect diagram of water with binder and thickener on the Mean viscosity at a shear rate of 10 s^−1^.

The interaction effects with water are shown in Figure 7.

Both effects have a level of significance greater than 0.1 (0.16, 0.15) and are therefore not statistically significant. Nevertheless, they both show the same interesting effect. At higher additive contents, the decrease in viscosity becomes more significant with increasing water content.

### 3.2. Macrostructure—Foam Cells

**Figure 8:**
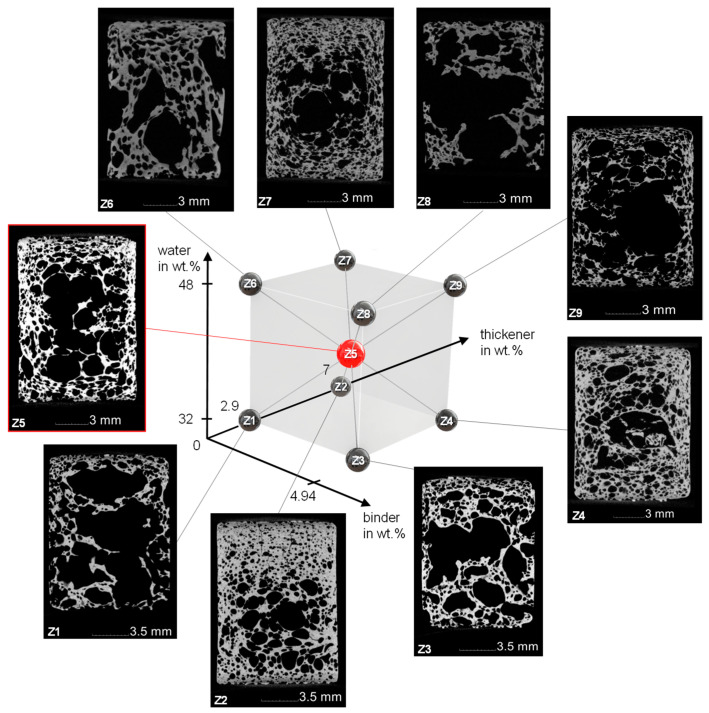
Structure of the DoE with corresponding CT images of the sintered Freeze Foams.

Sintered freeze foams were examined with CT-analysis. Cutting planes in the middle of the examined cylindrical foams including the structure of the DoE are presented in Figure 8. The corresponding foam cell porosity (*FCP*) is shown in Table 2.

The *FCP* value ranges from 40.5% for Z2 (water and binder ↓, thickener ↑) to 72% for Z8 (water and binder ↑, thickener ↓). The cutting planes of the different foams show a large variety of foam cell morphologies. Common to all foams are the large foam cells in the middle as well as a densification at the lateral surface where the rubber mold hindered the expansion and at the primary surfaces where the sieves stopped further foam growth. Although the sieves should allow pore forming gases to escape when cell walls are torn, it seems this did not occur fast enough, so the resulting pressure increase inside the foam led to the growth of very large foam cells. It should also be mentioned that due to the limited resolution of 28.3 µm voxel size, very thin struts may not be visible in the CT scans. A more detailed examination of these different morphologies was not part of this study.

**Figure 9:**
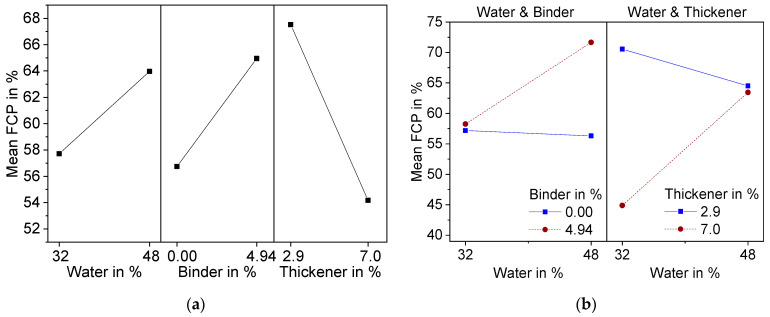
Effect plots of foam cell porosity (*FCP*) resulting from DoE; (**a**) Main effects plot; (**b**) Interaction effect plot.

The statistically significant factorial effects after backwards elimination are presented in Figure 9 as a main effect plot (a) and interaction plot (b).

Variation of thickener content showed the strongest effect in the form of a lowered *FCP*, which can be explained by the increase in viscosity due to the thickening mechanism of TAFIGEL AP15. Measurements of shear viscosity have proven this (Figure 6). Significant two-factor interactions were found for water–binder and water–thickener. For both, the porosity of the foam cells increases with increasing water content, but only at high binder or thickener contents. This can be explained by the more pronounced decrease of viscosity at higher additive content (Figure 7), although it must be stated that these findings are not statistically significant. Another possible explanation is the effect of polyvinyl alcohol to reduce the surface tension of water, thereby stabilizing air bubbles insight the suspension. That is why suspension with higher binder content might contain more air, which leads to larger foam growth. Moreover, a higher water content is associated with increasing amount of thickener (Equation (2)) so that viscosity becomes less dependent on the water content.

### 3.3. Microstructure—Strut pores

The strut porosity (*StP*) of sintered freeze foams was calculated according to Equation (3), based on the values listed in Table 3. The bulk density of sintered freeze foams is 3.1 g/cm^3^. *StP* values reach from 10.3% for Z2 to 36.5% for Z8. The results of the DoE are shown in Table 3.

Significant factors are water and thickener and their interaction. Although the addition of binder leads to significant differences in pore morphology, especially at high water content, it is not significant with respect to strut porosity. Variance analysis resulted in a *p*-value of 0.53, which is above the chosen level of significance of 0.1. The hindrance of ice crystal growth is probably compensated for by an increase in pore volume due to burnout during thermal treatment. An increase in water content has the highest effect on *StP*, because water is a pore forming factor besides burned-out organic material. Higher amounts of thickener lead to a decrease in *StP*. It can be assumed that the increase in viscosity caused by thickener is responsible for this effect.

**Figure 10:**
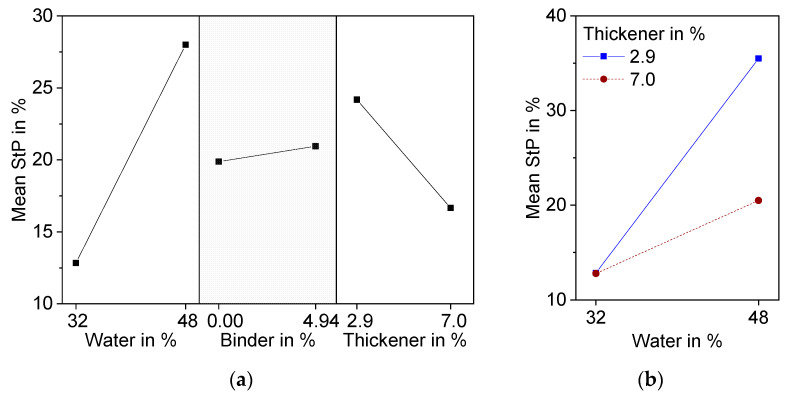
Effect plots of Strut Porosity (*StP*) determined with equation 3 resulting from DoE; (**a**) Main effects plot; (**b**) Interaction effect plot.

**Figure 11:**
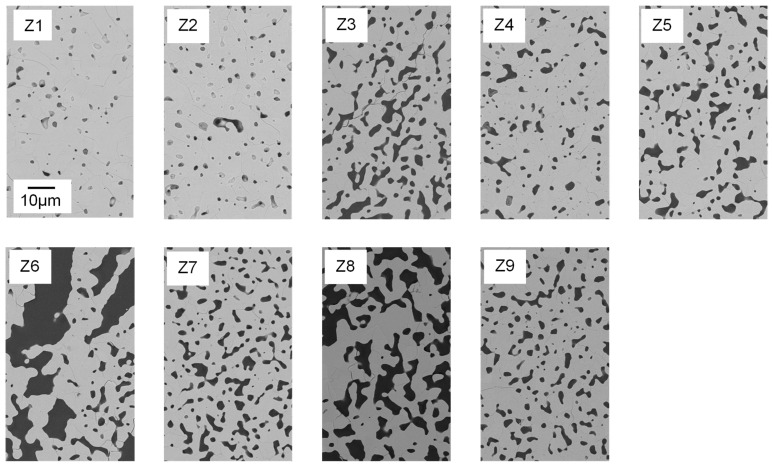
Polished cross-sections of Freeze Foams Z1–Z9, each at 1000× magnification recorded by electron microscopy (FESEM). The scale bar applies to all depicted cross-sectional images.

Water and thickener show significant interaction effects (Figure 10b). At low water content, increasing the amount of thickener resulted in very little decrease in *StP*, while the decrease in *StP* was much more pronounced at high water content. An explanation for this can be found in the cross-section images of the microstructure detailed in Figure 11. At 32 wt.% water, even at low binder and thickener contents, only small globular pores can be found (Z1). Therefore, in this case, an increase of thickener has no effect on freeze structure formation and thereby on strut porosity (Z2).

Strut pore morphology and pore size were characterized by means of FESEM and Mercury porosimetry. In Figure 11, FESEM images of foam struts from the nine different suspension compositions are displayed. Analogous to the foam cells, the struts also exhibited a large variety in pore morphology and size.

**Figure 12:**
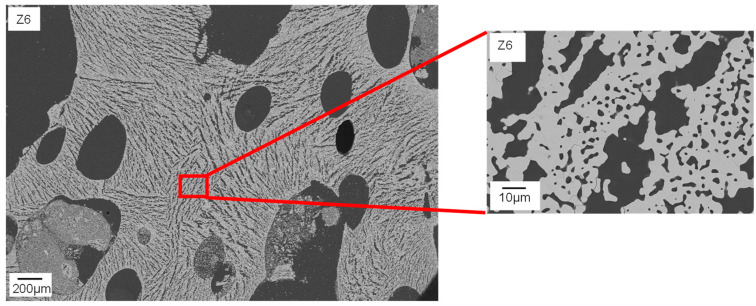
FESEM image of the microstructure of a sintered Freeze Foam Z6. Struts show distinctive freeze structures.

The shape of the pores ranges from globular (e.g., Z1, Z2) to interconnected channel pores (e.g., Z8), as displayed in Figure 11. Only Z6 (water ↑, binder and thickener ↓) has a different strut pore morphology, exhibiting significantly larger pore sizes. Figure 12 clarifies that the struts of Z6 show typical freeze structures commonly known from freeze casting [16]. The combination of high water content and low viscosity seems to allow the growth of larger ice crystals during pressure reduction. Smaller globular pores between the lamellar pores (Figure 12, right) can probably be referred to burned-out organic material.

In the struts of Z8, no lamellar pores are observed, although the suspension contained 48 wt.% water and displayed low viscosity. In contrast to Z6, it contained binder. Therefore, it can be concluded that binder molecules may have a large influence on ice crystal growth and thereby on strut pore morphology. This confirms the findings of Dedovets and Deville [30]. Furthermore, in the struts of Z7 (water ↑, binder ↓, thickener ↑), no freeze structures are observed as well. In this case, the thickener content of the suspension was higher compared to Z6 (7 wt.%). Here, it is assumed that the increased viscosity of the suspension hinders the ice crystal growth and subsequently leads to smaller and more globular shaped, partly interconnected pores. Composition Z1 contains no binder and only 2.9 wt.% thickener, resulting in similar rheological properties to Z6. The only difference is the lower water content (32 wt.%). This leads to smaller particle–particle distances and therefore blocking of growing ice crystals, as stated by Naglieri et al. [31] and recently confirmed by Schelm et al. [32] and Dammler et al. [33].

**Figure 13:**
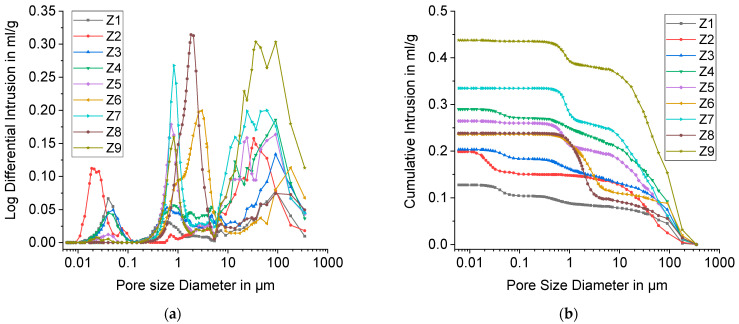
Mercury porosimetry results for DoE points Z1 to Z9; (**a**) Log Differential Intrusion of mercury; (**b**) Cumulative Intrusion of mercury against the Pore size Diameter.

The results of Mercury porosimetry are shown in Figure 13.

The pore size distributions display up to three peaks. The one at the far right reflects foam cells. Its range encompasses 5–400 µm with the exception of Z6, for which it starts at around 10 µm. These pore sizes can be attributed to the largest strut pores respectively. With Mercury porosimetry, only pores with a size smaller than 400 µm can be observed. For this reason, only a part of freeze foam porosity can be detected with this method and larger foam cells were analyzed separately with CT (Section 3.2).

The middle peaks (2nd peak) in the pore size distribution starting at approximately 0.1 µm represent open strut pores. Table 4 shows the position of the 2nd peak. It has to be noted that with Mercury porosimetry only the size of pore openings is determined. For Z2, no distinctive 2nd peak was observed. Presumably, strut pores are mainly closed and not accessible for mercury. Moreover, the peak is much more pronounced for higher water contents (40, 48 wt.%), which means that there is a higher fraction of open and interconnected pores in the struts.

The median pore size (2nd peak) reaches from 0.5 to 0.8 µm with the exception of Z6 (3.0 µm) and Z8 (1.8 µm). These values are in good accordance with FESEM images in Figure 12.

**Figure 14:**
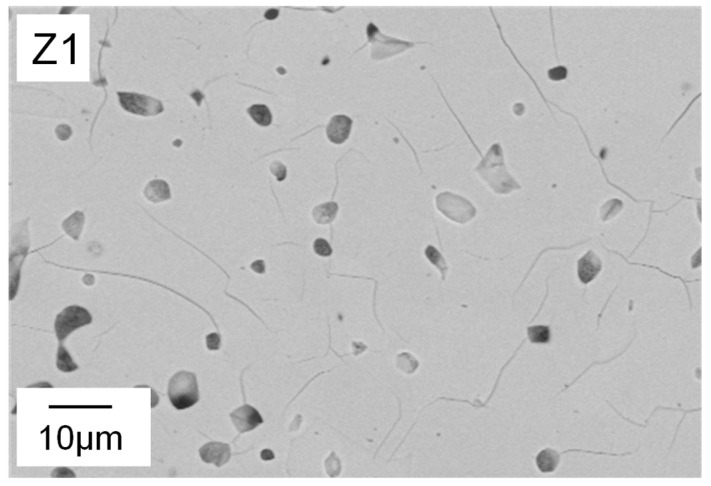
Polished cross-sections of Freeze Foam Z1 at 1000× magnification recorded by electron microscopy (FESEM) showing pores that are connected by cracks.

Interestingly, a third peak appeared between 0.01 and 0.1 µm, especially for foams with low water content. This is probably due to cracks that connect closed strut pores (Figure 14).

The area beneath the third peak represents the crack volume and the closed strut pore volume, which are detectable by Mercury porosimetry. Therefore, it can be concluded that closed *StP* is lowered with increasing water content, because only foams Z1–Z4 with 32 wt.% water exhibit a pronounced peak below 0.1 µm. As an alternative theory, the authors propose that there are open intergranular pores between the primary particles. At higher water contents, the ice crystals grow larger and force the HAp particles into denser packaging. This would result in smaller intergranular pores which disappear during sintering.

### 3.4. Model Suspension

In the sections above, the influence of the suspension parameters on the resulting freeze foam structure was investigated in detail. The following aim was to develop a composition that allows stable foaming with foam cell porosity (*FCP*) around 60% by varying water content. With this model suspension, the influence of additional, important process parameters such as pressure reduction rate on the foaming behavior should be investigated. The following model equation resulting from the DoE was used to determine the optimal composition of the model suspension. The target factor is the *FCP*:

*FCP* = 148.4 − 1.914 × *water* − 5.57 × *binder* − 18.27 × *thickener* + 0.1807 × *water* × *binder* + 0.3755 × *water* × *thickener* (4)

A binder content of 1.3% was found to be well suited in combination with 4% thickener to guarantee stable foaming behavior and good demolding capability. Less binder would also be possible, but this leads to a reduced stability of the green foams. The predicted values for *FCP* for three different water contents are shown in Table 5.

In a water content span of 14 wt.%, the *FCP* only differs by about 2.5%. The possibility to vary the water content while only slightly influencing the *FCP* allows for adjusting the *StP*, which influences for example the shrinkage during sintering. This allows to combine the high biocompatibility of the Freeze Foams [21] with the constructional freedom of additive manufacturing approaches like lithography-based ceramic manufacturing (LCM) to create patient specific bone implants with improved compressive strength [18]. Recently, Ahlhelm et al. used additively manufactured structures to be filled in green state with freeze foam and co-sintered both to a β-TCP hybrid structure that showed high biocompatibility [34]. In the ongoing BMBF-funded project “Hybrid-Bone” (03VP07633), this hybrid shaping method is used to develop compressive strength-enhanced, biodegradable jaw-bone replacements.

Moreover, as stated above, a high strut porosity and even freeze structures in the struts are possible mainly at high water contents. These can be beneficial for bone replacement materials. That is why suspension with 48 wt.% water content was chosen for the following investigation of the foam growth.

### 3.5. Results of Radiographical and CT Evaluation of the Foam Growth

**Figure 15:**
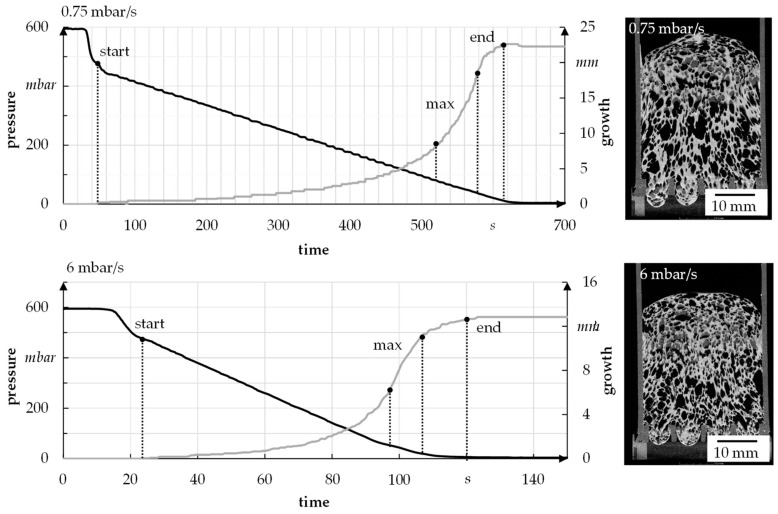
Sample for growth of foam in relation to pressure reduction rate.

To analyze the foam growth and structure formation via radiographic investigation, the model suspension with 1.3 wt.% binder, 4 wt.% thickener, and 48 wt.% water was used. It was foamed at different speeds and then scanned. The suspension was initially foamed with a pressure reduction rate of 6 mbar/s (quick) and 0.75 mbar/s (slow) to determine the limits, i.e., the start and end of foaming, of a foaming process (see Figure 15). In each case, six samples were used to perform the tests. For the used suspension, foaming starts at about 470–490 mbar and ends at 7–10 mbar, independent of the foaming rate. The fastest foam growth takes place in the range of 30–100 mbar.

**Figure 16:**
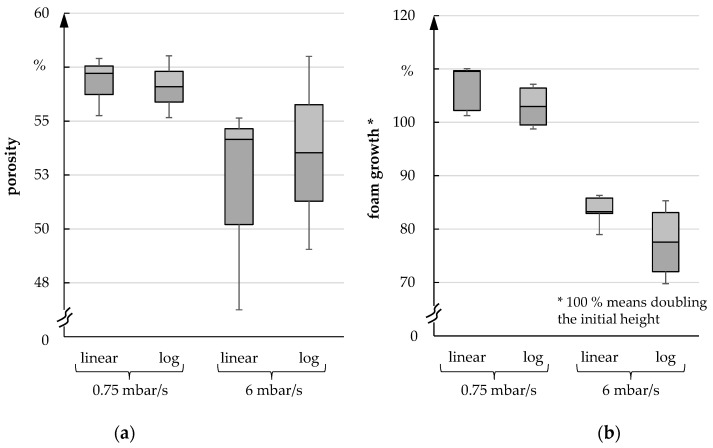
(**a**) Porosity; (**b**) Foam growth for 0.75 mbar/s und 6 mbar/s.

After analyzing the foaming process, the porosity and the foaming height of the foam were investigated. In each case, the pressure was reduced linearly as well as logarithmically since the freeze dryer ALPHA 2-4 LSC PLUS lowered the pressure logarithmically during the freeze foaming process. However, a linear pressure reduction allows a better analysis of the foam structure formation. In the following figures, both types of pressure reduction are compared with each other. A difference between linear or logarithmic pressure reduction could not be detected (median deviates less than 1% and can be ignored). Figure 16a shows that the porosity exhibits clear differences between fast and slow pressure reduction. The porosity is approximately 4–5% lower after a fast pressure reduction. Nevertheless, the foam volume is significantly higher under these conditions compared to the fast-pressure reduction scenario. Similarly, a significantly higher height of up to 20% can be observed with a slow pressure reduction (Figure 16b).

**Figure 17:**
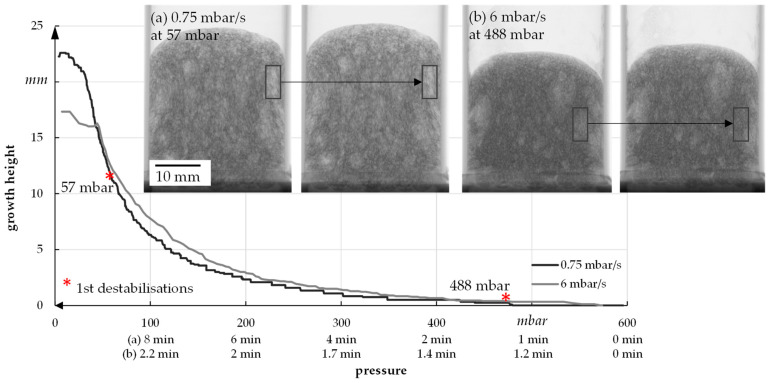
Foam growth as a function of pressure.

For a better understanding of the growth process, the foam growth is shown as a function of pressure for one sample (see Figure 17). In general, it has already been established that a slow reduction of the pressure leads to increased foam height as well as porosity.

The example radiographic images in Figure 17b show that at fast pressure (6 mbar/s) reduction, an exemplary foam cell (marked with a square) collapses at 488 mbar. This indicates the rupture of cell walls that leads to escaping air and water vapor and therefore inhibits further foam growth. For a slow pressure reduction, the first foam cells start collapsing at 57 mbar, which leads to a much higher foam growth but also larger foam cells (Figure 15). In general, the first destabilization effects appeared between 200 and 500 mbar at fast pressure reduction and between 30 and 70 mbar at slow pressure reduction. A slower pressure decrease also allows the system more time for water vapor production and attaining equilibrium conditions. The more water vapor, the higher the driving force for foam volume growth. However, at slow pressure decrease, the time until consolidation of the foam structure happens is longer compared to a fast decrease. That is why coalescence and Ostwald ripening are increased. Both effects lead to an inhomogeneous foam structure, which can be seen in Figure 15. To manufacture a homogeneous freeze foam with high foam cell porosity, it will be necessary to find an optimum pressure reduction rate that allows high foam growth but prevents destabilizing effects.

## 4. Conclusions

In the first part of this study, the influence of the suspension composition, including water, binder, and thickener contents, on the resulting macro- and microstructure of sintered freeze foams was investigated. All suspension were foamed at room temperature (23 °C). Macrostructural analysis was conducted with an in-situ radiographical CT device and the microstructural analysis with FESEM and Mercury porosimetry. Macrostructural foam cells grow during pressure reduction in the freeze dryer because of inflating air inside the suspension and water vapor. In contrast, microstructural strut pores are mainly replica structures of frozen water, so their morphology depends on ice crystal growth. The main findings were:

1. The foam cell porosity (*FCP*) is mainly dependent on the thickener content. The mean value reaches from 68% to 54% for 2.9 and 7 wt.% thickener in relation to water content.
2. Water content has the largest effect on strut porosity (*StP*). Mean values for *StP* with rising water content increase from 10.3% to 36.5%. The addition of thickener lowers the *StP* significantly, meaning it can be tuned by adjusting water and thickener contents.
3. The morphology of the strut pores is largely dependent on the binder content. Without binder and at low thickener and high water contents, the formation of lamellar freeze structures was observed. Thus, it is now possible to adjust the strut pore morphology in a way that can be useful, e.g., for fluid or gas transport.

Using a model equation for *FCP* that resulted from the conducted DoE, a model suspension was developed with optimal foaming behavior. Based on this suspension, the foaming behavior was monitored using an in-situ CT device, which was developed and built specifically for this purpose. The investigations have shown that foam cell walls remain stable until lower pressure in the slower foaming process, while foam destabilizing effects, such as coalescence and Ostwald ripening, are increased at the same time. This leads to higher foam cell porosity but a more inhomogeneous structure at a slow pressure reduction rate. To find an optimum, these effects need to be investigated in more detail in the further course of this project. With the acquired knowledge, the macrostructure and the microstructure of freeze foams can now be tuned independently for the first time, and therefore a tailoring to specific applications like bone replacement material for different bone regions is possible. Further work shall focus in greater detail on manipulating the pore morphology of both macro- and micropores.

## Figures and Tables

**Table 1:** Two-stage full factorial Design of Experiments with three factors and central point.

Suspension	Run Order	Central Point	Water in wt.%	Binder in wt.%	Thickener in wt.%
Z7	1	1	48	0.00	7.00
Z6	2	1	48	0.00	2.90
Z5_1	3	0	40	2.47	4.95
Z5_2	4	0	40	2.47	4.95
Z8	5	1	48	4.94	2.90
Z4	6	1	32	4.94	7.00
Z2	7	1	32	0.00	7.00
Z9	8	1	48	4.94	7.00
Z3	9	1	32	4.94	2.90
Z5_3	10	0	40	2.47	4.95
Z1	11	1	32	0.00	2.90

**Table 2:** Foam cell porosity (*FCP*) of sintered Freeze Foams at the points of the DoE. For Z5 three foams were measured with a standard derivative of 0.9%.

	**Z1**	**Z2**	**Z3**	**Z4**	**Z5**	**Z6**	**Z7**	**Z8**	**Z9**
***FCP* in %**	73.9	40.5	67.2	49.3	57.3	57.0	55.6	72.0	71.3

**Table 3:** Foam weight *m*_foam_, strut volume *V*_strut_ and calculated strut porosity *StP* for sintered Freeze Foams at the different design points of the DoE.

Design Point (Water-Binder-Thickener)	*m*_foam_ in g	*V*_strut_ in cm^3^	*StP* in %
Z1 (32-0-2.9)	1.2799	0.469	11.9
Z2 (32-0-7)	2.9308	1.054	10.3
Z3 (32-4.94-2.9)	1.5276	0.572	13.8
Z4 (32-4,94-7)	2.2250	0.847	15.3
Z5 (40-2,47-4.94)	1.6770	0.669	19.2
Z6 (48-0-2.9)	1.2117	0.596	34.5
Z7 (48-0-7)	1.5442	0.645	22.8
Z8 (48-4.94-2.9)	0.7752	0.394	36.5
Z9 (48-4.94.-7)	1.1027	0.435	18.2

**Table 4:** Median size of strut pore openings determined by the position of the 2nd peak in the pore size distribution (Figure 13).

Design Point (Water-Binder-Thickener)	Pore Size 2nd Peak in µm
Z1 (32-0-2.9)	0.5
Z2 (32-0-7)	-
Z3 (32-4.94-2.9)	0.6
Z4 (32-4.94-7)	0.8
Z5 (40-2.47-4.94)	0.7
Z6 (48-0-2.9)	3.0
Z7 (48-0-7)	0.8
Z8 (48-4.94-2.9)	1.8
Z9 (48-4.94.-7)	0.8

**Table 5:** Predicted values for ***FCP*** according to Equation (3). The value for binder was set to 1.3 and for thickener to 4.0.

Water Content	34 wt.%	41 wt.%	48 wt.%
Predicted *FCP* in %	62.1 ± 2.0	60.8 ± 1.6	59.6 ± 2.4

## Data Availability

Data are included in the article.
